# A case of rapidly progressing and poorly differentiated Ip‐type early‐stage colorectal adenocarcinoma

**DOI:** 10.1002/deo2.181

**Published:** 2022-10-26

**Authors:** Atsuko Tsubomoto, Hiroshi Sashiyama, Junichi Koike, Yohei Morita, Osamu Tsutsumi, Yukihiro Hamahata

**Affiliations:** ^1^ Department of Colorectal Surgery Tsujinaka Hospital Kashiwanoha Chiba Japan

**Keywords:** early‐stage cancer, implantation, Ip‐type, Ki67 antigen, poorly differentiated colorectal adenocarcinoma

## Abstract

A 36‐year‐old woman visited our hospital with a chief complaint of bleeding during defecation. Colonoscopy revealed a 20‐mm pedunculated polyp in the sigmoid colon, which was en bloc resected under endoscopy. The histopathological diagnosis was adenoma cancer with a depth of invasion indicating mucosal cancer, no lymphovascular invasion, and negative at the resection margin. The poorly differentiated adenocarcinoma component comprised approximately 5% of the tumor. Although there were no recurrence signs in the computed tomography scans obtained 4 months post polypectomy, the patient experienced aggressive lower back pain at 6 months post polypectomy. Local recurrence, peritoneal dissemination, and liver metastasis were confirmed. Finally, the patient died following a rapid and aggressive deterioration of her general condition. Histological examination of the local recurrence revealed a poorly differentiated adenocarcinoma (por2), with immunostaining revealing a high Ki67 positivity rate of 95%. Moreover, the poorly differentiated adenocarcinoma region of the resected polyp had a Ki67 positivity rate of 90%, which suggested that they were the same tumors. These findings suggested that the recurrence could have occurred through implantation.

## INTRODUCTION

Early‐stage colorectal cancer with poorly differentiated adenocarcinoma is a rare condition, with many cases showing a depth of invasion of submucosa and lymphovasculature. Most of these patients are treated through surgery. We report a case of intramucosal cancer with a pedunculated poorly differentiated adenocarcinoma component with recurrence.

## CASE PRESENTATION

**FIGURE 1:**
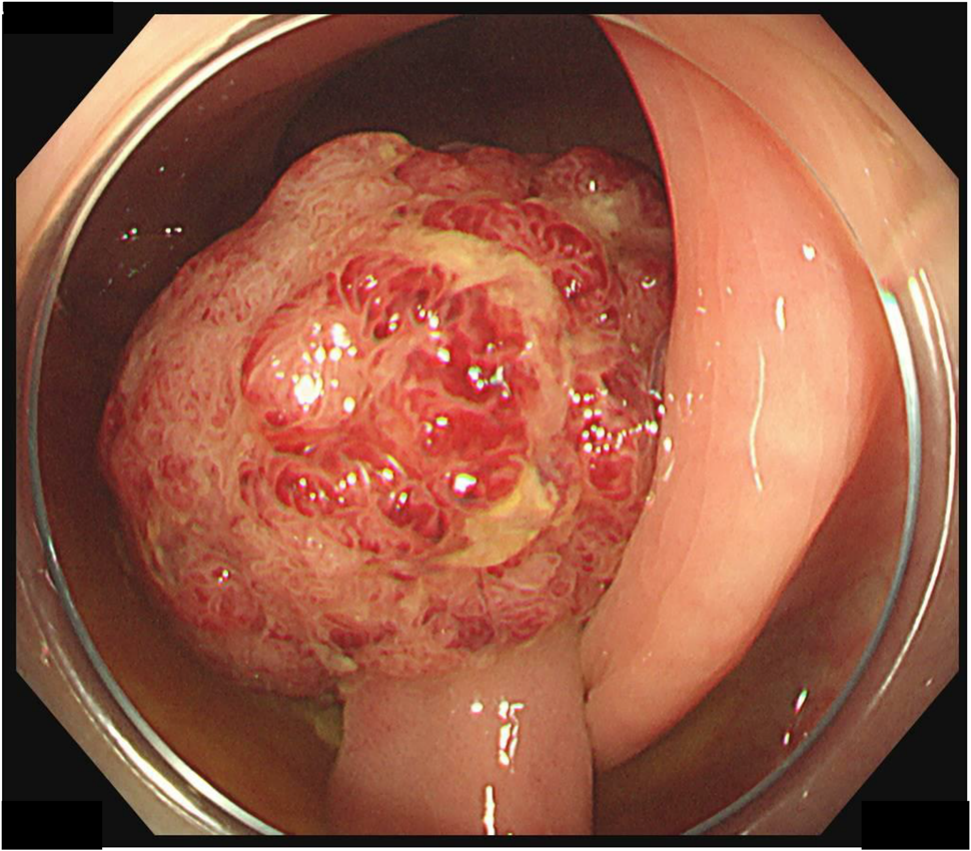
Colonoscopy revealed a 20‐mm pedunculated polyp in the sigmoid colon. There was no tension in the stem; further, there was redness and adhesion of white mucus to the apex of the ridge

**FIGURE 2:**
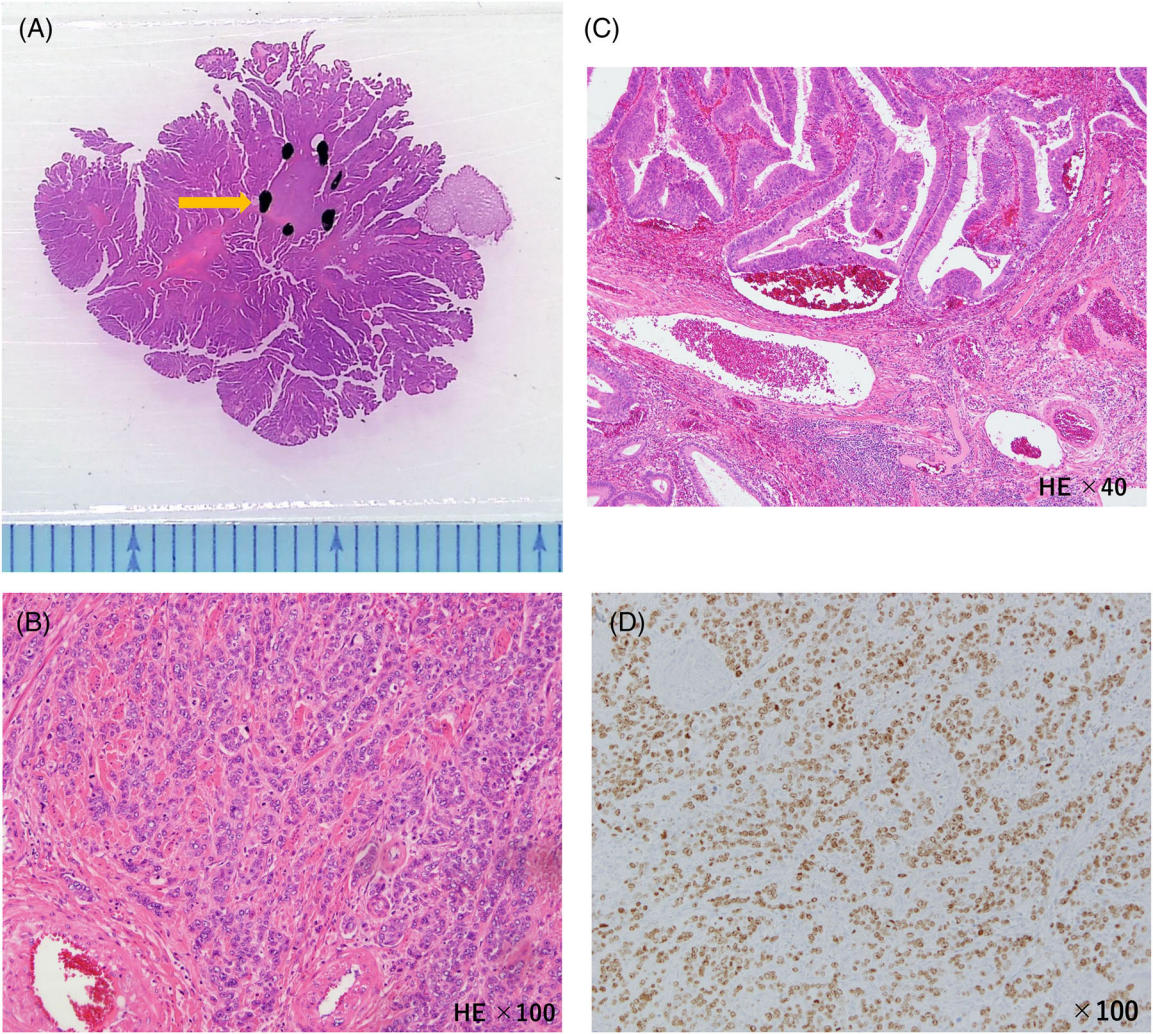
Histopathological examination of the polyp specimen. (a) Hematoxylin and eosin (HE)staining. Magnified image. The section indicated by the arrow is the poorly differentiated adenocarcinoma area, which accounted for approximately 5% of the tumor. (b) HE staining. Magnification of 100‐fold. There was a poorly differentiated component with a cord‐like structure and indistinct duct formation. (c) HE staining. Magnification of 40‐fold. There was no lymphovascular infiltration. (d) Ki67 staining. Magnification of 100‐fold. The Ki67 positivity rate in the poorly differentiated adenocarcinoma component of the polyp specimen was 90%

**FIGURE 3:**
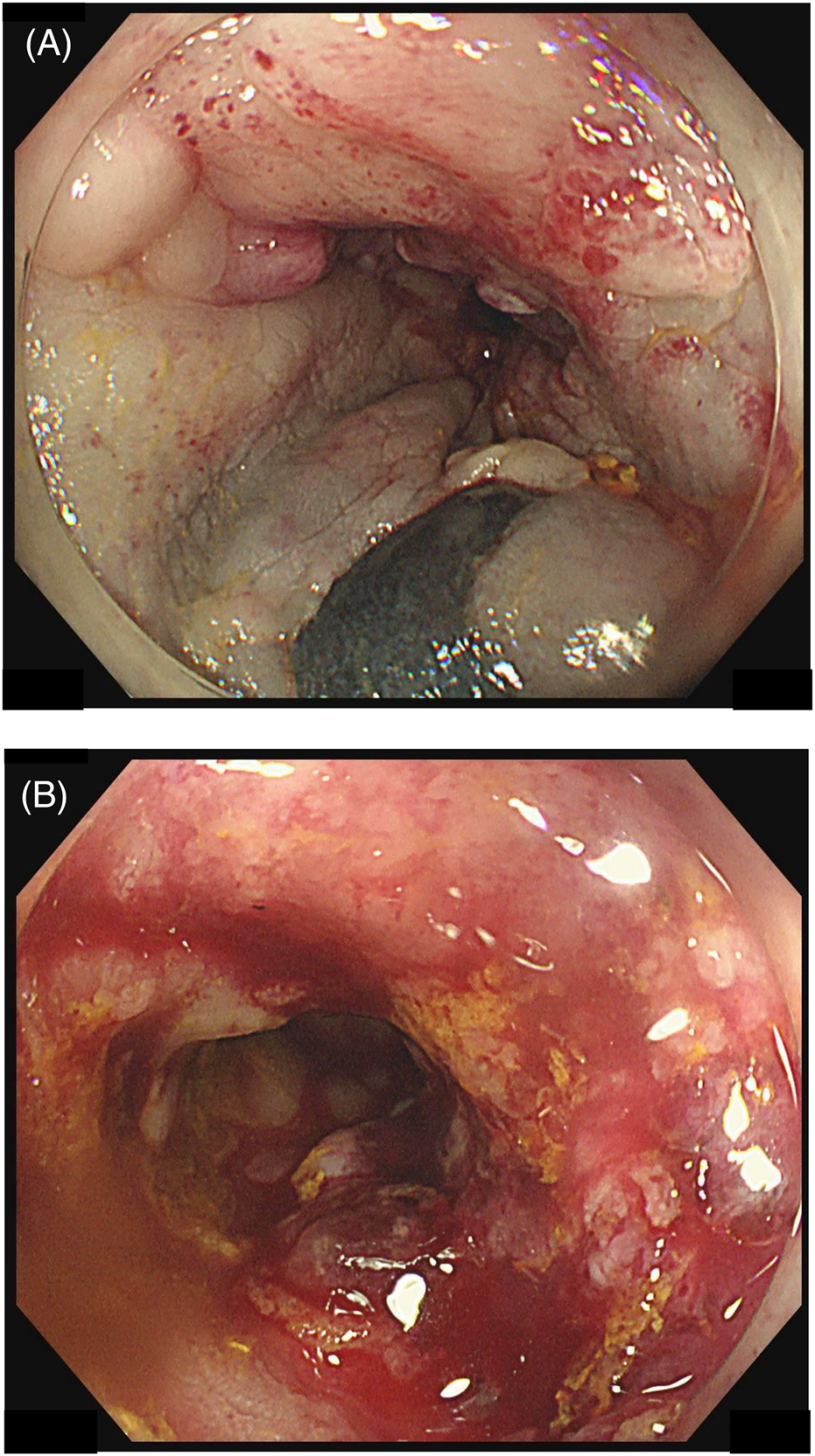
(a and b) Colonoscopy upon recurrence. (a) There was an irregular ulcerative lesion without a subcircumferential perimeter on the oral side of the tattooing. The mucosa was partially reddish and could easily bleed. Further, the scope barely passed through the lumen; however, there was stenosis over a major axis length of 50 mm. (b) Biopsy site

**FIGURE 4:**
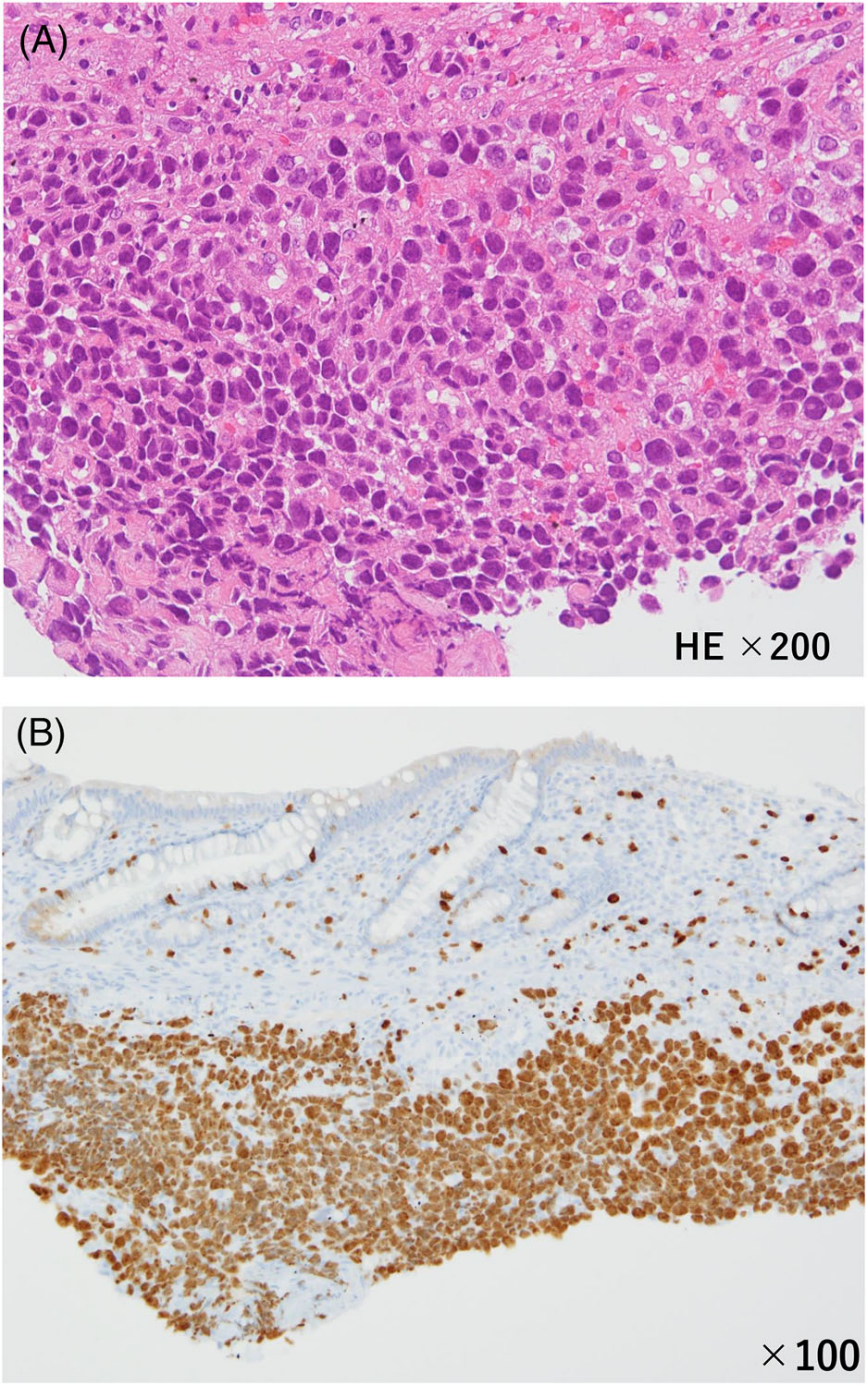
(a and b) Histopathological examination of the recurrence biopsy site. (a) Hematoxylin and eosin (HE) staining. Magnification of 200‐fold. Atypical cells with a high nuclear‐to‐cytoplasmic ratio showed scattered proliferation. Moreover, we observed a poorly differentiated adenocarcinoma. (b) Ki67 staining. Magnification of 100‐fold. The Ki67 positivity rate was >95%

A 36‐year‐old woman visited our hospital with a chief complaint of bleeding during defecation for several months. Colonoscopy revealed an Ip polyp (Figure 1) with a horizontal axis diameter of 20 mm and a length of 20 mm, a slightly nodular uneven surface and redness, and white mucus adhesion on the sigmoid colon with an anal verge of 25 cm; there was no tension in the stem. The patient underwent en bloc hot snare polypectomy; however, the tumor was fragile and fragmented upon removal through the anus. Extensive tumor resection was attempted. Histopathological examination revealed adenoma cancer, with the poorly differentiated adenocarcinoma component comprising approximately 5% of the tumor (Figure 2a,b). There was no lymphovascular invasion (Figure 2c), and the resection margin was negative. The Ki67 positivity rate in the poorly differentiated adenocarcinoma component was 90% (Figure 2d). Post‐polypectomy thoracoabdominal CT scans revealed no lymph node or distant metastases. We performed colonoscopy again to confirm no residual tumor prior to ink tattooing of the anal side of the resection area. Since there was a poorly differentiated adenocarcinoma component, a second opinion was taken. Subsequently, with informed consent from the patient, it was decided that the patient would be carefully monitored without additional colectomy. Thoracoabdominal CT scans obtained at 4 months post‐polypectomy revealed no findings that suggested distant metastases or recurrence. However, the patient presented with severe lower back pain at 6 months post‐polypectomy. Furthermore, abdominal CT scans revealed peritoneal dissemination and ascites, which prompted urgent admission. Hematological examinations revealed anemia, a markedly elevated inflammatory response, and mild hepatic damage. Regarding tumor markers, there was a significant increase in anti‐P53 antibody levels. Contrast‐enhanced abdominal CT scans revealed an 84‐mm mass with an uneven contrast effect around the sigmoid colon; there was a small amount of ascites and multiple nodular lesions with a short axis length of 13 mm around the rectum. There was a low‐density area with a major axis length of 70 mm in the medial area of the left hepatic lobe. Colonoscopy revealed an irregular ulcerative lesion on the oral tattooing side without subcircumferential perimeter ridge (Figure 3a). The mucosa was partially reddish and could bleed easily. Further, the scope could barely pass through the lumen; however, there was stenosis over a major axis length of 50 mm (Figure 3b). Based on the histological examination, the patient was diagnosed with poorly differentiated adenocarcinoma (Figure 4a). The Ki67 positivity rate was >95% (Figure 4b). Three days after hospital admission, her respiratory and general condition showed continued deterioration. Thoracoabdominal CT scans obtained 5 days after hospital admission revealed no pleural effusion and pneumonia; there was a rapid increase in the ascites volume. The patient died 10 days after hospital admission.

## DISCUSSION

The case showed strong findings suggesting that the risk of cancer recurrence is positively correlated with the Ki67 positivity rate even for early colorectal cancer, which is not considered for colon resection. The success or failure of endoscopic treatment for early‐stage colorectal cancer as well as the posttreatment risk of residual recurrence and metastasis is determined through histopathological analysis of resected specimens. Local recurrence is determined based on the presence of tumor tissue/cells in the horizontal and vertical margins of the resected specimen, including the split resection. Further, lymph node metastasis is indicated based on submucosa infiltration ≥ 1000 μm (pT1b), lymphovascular invasion, histological subtype (poorly differentiated adenocarcinoma, signet ring cell carcinoma, mucoid carcinoma), and the presence/absence of tumor budding.^1^ Advanced cancer is not observed in type Ip polyps; furthermore, cancerous cases often present with adenoma cancer.^2^ Only 20% of patients present with submucosa infiltration and most of them present with a depth of invasion of up to T1a.^3^ Accordingly, these patients usually undergo endoscopic treatment. Recurrence after endoscopic resection occurs in 0%–4.2% of patients with colorectal M cancer.

Factors that determine local recurrence after endoscopic treatment for colorectal cancer include residual tumor at the resection margin, residual lymphovascular invasion, and implantation.^4^ The pathological finding of the resected polyp negative resection margin suggests that residual tumor does not exist at the resection margin. Therefore, we assumed that residual lymphovascular invasion and implantation could be a possible cause for the recurrence in this case. However, the recurrence was most likely caused by implantation due to the presence of free cancer cells in the intestinal tract, which could also cause recurrence at anastomotic sites in surgery. This is further supported by the clinical experience that cancer develops in the hemorrhoid resection wounds of patients with cancer.^5^ There have been several recent reports of cancer recurrence due to implantation in the wound after endoscopic submucosal dissection (ESD).^4,6^ Some patients with post‐ESD recurrence due to implantation may present with advanced cancer other than the lesion targeted with ESD. Here, cancer cells can be released by contact between the endoscope and tumor with subsequent engraftment of the cells in the ESD wound. Notably, free cancer cells engraft on the raw surface instead of normal mucosa.^6^

During the initial en bloc resection, the tumor fragmented, and free cancer cells in the intestinal tract could have engrafted at the resection margin, which led to cancer recurrence. Additionally, the high Ki67 positivity rate in the primary tumor could have contributed to the recurrence. The Ki67 positivity rates in the poorly differentiated adenocarcinoma component of the resected polyp and in the recurrent tumor were 90% and >95%, respectively. The Ki67 antigen, which is present in the cell nucleus during the late G1 to M phases, is a cell proliferation marker. Terabe^7^ reported an average Ki67 positivity rate of 38.5% in colorectal cancer. Funabashi et al.^8^ reported that the Ki67 positivity rates according to the histological subtype were as follows: well, 39.14 ± 13.26%; moderate, 48.83 ± 2.88%; signet, 59.85 ± 13.60%; and mucinous, 59.60%. The Ki67 antigen positivity rate is negatively correlated with the degree of differentiation. Moreover, the Ki67 positivity rate is significantly higher in colorectal cancer with serosal infiltration, venous invasion, lymph node metastasis, and distant metastasis than in colorectal cancer without these features. Additionally, the Ki67 positivity rate in the intramucosal area of patients with early‐stage depressed colorectal cancer is significantly higher than in patients with protruding early‐stage colorectal cancer. The Ki67 antigen positivity rate could be an objective indicator of a tumor's metastatic potential.^9^

The high Ki67 expression in our patient was suggestive of a high proliferative potential of the patient's cancer cells. This could have contributed to the short time to recurrence and the rapid deterioration of the patient's condition.

Lymphovascular recurrence may have also been a possibility. According to Japanese guidelines, pedunculated polyps are out of scope for an additional surgical procedure following endoscopical resection because the Japanese Society for Cancer of the Colon and Rectum reported that no lymphovascular recurrence was noted for pedunculated polyp without stalk invasion.^10^ However, some reports suggest that metastasis may occur with pedunculated polyps.^10^ In addition, even though this case had no risk factors apart from poorly differentiated type and high Ki67 rate, fragmented sample condition may affect pathological diagnosis. Therefore, patients with a pedunculated polyp without risk factors, as set by the above guidelines, could develop lymphovascular recurrence post‐polypectomy.

Although pTis and pedunculated cancer can be curatively resected through endoscopic treatment, Ki67 staining could be necessary for tumors containing por components. Additionally, when selecting subsequent treatment strategies, it is important to carefully consider the possibility of recurrence due to implantation. Although pTis cancer, a poorly differentiated adenocarcinoma, is rare, more case reports are required to establish the indication criteria for additional bowel resection.

This case of recurrent Ip‐type pTis cancer suggests that the risk of recurrence is positively correlated with the Ki67 positivity rate.

## CONFLICT OF INTEREST

The authors declare no conflict of interest.

## FUNDING INFORMATION

This research received no specific grant from any funding agency in the public, commercial, or not‐for‐profit sectors.
